# “We’ve got the home care data, what do we do with it?”: understanding data use in decision making and quality improvement

**DOI:** 10.1186/s12913-020-5018-9

**Published:** 2020-03-25

**Authors:** Jacobi Elliott, Alicia Gordon, Catherine E. Tong, Paul Stolee

**Affiliations:** School of Public Health and Health Systems, University of Waterloo, 200 University Avenue West, Waterloo, Ontario N2L 3G1 Canada

**Keywords:** Decision-support, interRAI data, Education, Home care, Quality of care, Safety, Standardized assessments

## Abstract

**Background:**

In the past decade the provision of home care services in Canada has doubled; with this increase there has been a growing interest in improving quality and safety. National-level data are captured in the Home Care Reporting System (HCRS), using the interRAI-HC tools. Data in the HCRS provide decision-makers and administrators with actionable evidence to inform quality and safety improvement initiatives. The objective of this study was to determine how providers and administrators were using these data, and if there were any opportunities to enhance their use.

**Methods:**

We collaborated with the Canadian Patient Safety Institute to conduct this qualitative study. To understand data use in home care, we recruited participants in management and/or data analysis roles from home care organizations across Canada. We conducted three individual interviews and three focus group interviews with a total of eleven participants from five Canadian provinces. Individual and focus group interviews were audio recorded, transcribed, and analysed using a combination of emergent coding and thematic analysis.

**Results:**

Several participants provided powerful examples of how interRAI data have been used to guide patient safety and quality improvement initiatives; however, all participants recognized challenges in using these data. Leveraging interRAI data in the complex home care environment is limited by several factors: the general “newness” of these data in many jurisdictions; the sheer volume of data; limited capacity and resources to interpret and analyse the data; and connectivity issues in rural areas. Participants recognized and appreciated the training that has been provided, and made several recommendations for additional training.

**Conclusions:**

Mandated clinical datasets, such as the suite of interRAI tools, have the potential to improve quality and inform decision-making. However, in order to utilize these data, home care agencies require additional education, personnel and supports. Additional training and resources for these organizations could improve the use of available data by home care agencies, as well as outcomes for individuals receiving home care services.

## Background

Individuals with chronic health conditions or disabilities overwhelmingly indicate a desire to remain in their home as long as possible, regardless of their condition [1]. Home care has been cited as a preferable method of care delivery for individuals with chronic, palliative, or rehabilitative needs due to the sense of retained independence and familiarity [1]. This preference, coupled with rising health care costs, has led to a push to reduce long term care (LTC) and acute care admissions, and increase home care service provision to more efficiently utilize the limited healthcare resources in Canada [2]. Funding and provision of home care services varies across Canada. Each province and territory administers its own publically-funded home care services, which vary in terms of coverage, eligibility criteria, and which services are partially or fully funded [2]. In 2012, 2.2 million people received home care in Canada; 40% of those were aged 65 and older [1]. In the past decade, the provision of home care services has doubled, and this growth is projected to continue with the aging population [3].

The increase in home care service provision has resulted in a growing interest in improving the safety and quality of home care [3]. The Canadian Foundation for Healthcare Improvement defines quality improvement as “a sustained effort to improve healthcare quality that incorporates repeated performance measurement and feedback to healthcare providers” [4] [p6]. This concept is increasingly important for the health sector as it ensures that, with advancements in technology, knowledge, and practice, the core focus is providing the best, and safest, care possible. However, the differences in provision models across the country pose a challenge in ensuring and monitoring a uniform standard of care. Variations in provision models make pan-Canadian comparisons of home care services difficult, limiting the implementation of safety and quality improvement initiatives. Safety in home care is a critical area of concern: a pan-Canadian study on home care safety found that 56% of adverse events were judged to be preventable and 68.8% resulted in a disability [3].

One opportunity for capturing national-level data is the Home Care Reporting System (HCRS), developed by the Canadian Institute for Health Information (CIHI). The HCRS database stores demographic, clinical, functional, and resource utilization data for patients who receive care from publicly-funded home care programs across Canada [5]. Data in the HCRS are captured from several sources across Canada, including ministries of health and regional organizations, using interRAI tools including the interRAI Home Care Assessment (interRAI-HC© [6]) and the interRAI Community Health Assessment (interRAI-CHA© [7]). interRAI instruments are standardized measures that are widely used and largely mandated in Canada, allowing for pan-Canadian comparisons of home care data [5].

In home care, interRAI data are collected by designated assessors, registered healthcare providers, who have received training on the administration of the tools. interRAI-HC captures comprehensive patient data, including cognition, service utilization, social support, physical function, diseases and disease prevention, nutrition, hearing, vision, etc. [8]. interRAI data can be used to guide patient-level clinical decision-making and organization-level quality improvement initiatives [8, 9]. For example, data from the interRAI HC and CHA can be used to score home care quality indicators (HC-QIs). HC-QIs assess organizational performance across a range of domains, including functional, clinical, social and service quality indicators [9]. Currently, the HCRS database contains data from seven of the ten Canadian provinces, and one of the three Canadian territories [5].

Data in the HCRS provide decision-makers with actionable evidence to inform quality and safety improvement initiatives. Despite the availability of this resource, information on interventions informed by these data are limited, suggesting initiatives to improve the understanding and use of RAI data in home care settings are warranted. The purpose of this project was to determine how home care managers and providers were using home care data (both RAI-based data and other locally collected data) to address safety issues and implement quality improvement initiatives, and whether there were any opportunities for provision of further education or other resources to support effective use of the data.

Specifically, we wanted to learn from home care managers and providers:

1. What their experiences were with using home care data (e.g., their confidence and ability in using data, accessibility of data, process)
2. How the data are being used for addressing safety issues.
3. How the data are being used for implementing quality improvement initiatives.
4. Current education received for using these data.
5. Ideas for materials or resources that could be made available to increase knowledge.

This study focuses on understanding data use at the organizational and provincial level, not at the point of care.

## Methods

Our university-based research team collaborated with the Canadian Patient Safety Institute’s (CPSI) Theme 4 Action Committee to carry out this qualitative study. CPSI was keen to understand how data are being used within the home care sector, and how these data are, or could be, leveraged to improve patient safety and quality of care.

### Recruitment & Participants

To understand data use in home care, we sought to interview individuals in management and/or data analysis roles from agencies and providers across the country. The participants were recruited from provincial home care agencies or health authorities. Members from the CPSI Theme 4 Action Committee invited prospective participants with a letter of information. Participants were first approached by CPSI, who obtained the participant’s permission to be contacted by the research group. CPSI approached individuals who would be able to comment on data use at organizational and provincial levels, educational requirements, and data use for quality improvement initiatives. Upon agreement, an interviewer from the research group called the participant and obtained verbal informed consent. We also obtained written informed consent via email, prior to the interview. Eleven individuals from five different provinces agreed to participate: one in British Columbia, five in Alberta (representing three organizations), one in Saskatchewan, two in Manitoba (representing one organization), and two in Ontario (representing two organizations). Participant roles included: Manager of Quality and Innovation; Director of Quality Assurance and Risk; Executive Director for Continuing Care; and Director of Quality Risk Management, Client Care Manager, Project Director for Seniors Mental Health and Addictions, Researcher, and Program Consultant.

### Data Collection & Analysis

We conducted three open-ended semi-structured telephone interviews, and three telephone focus group interviews (*N* = 11). Each interview/focus group lasted approximately 1 h, and was conducted by an experienced qualitative facilitator (JE) with in-depth knowledge of the home care sector. The interview/focus group guide was developed in consultation with CPSI, and sample questions are included in Table 1. The complete interview guide is available as supplementary documentation. Research assistants joined the group calls as note-takers. Interviews were audio recorded and transcribed verbatim. Two team members who participated in the interviews (JE, AG), and one who did not (CT), were involved in the analysis. Transcripts were first analyzed through line-by-line directed coding, followed by emergent coding with JE and AG using NVivo 11 software [10]. Thematic analysis was used to derive themes and sub-themes [11]. Finally, a team member with experience in the home care sector (CT) conducted a final review of the transcripts and synthesized the themes and sub-themes into the results presented here. Team-based analysis, the thoughtful examination of outliers, and memoing enhanced the rigour of the analysis [12].

**Table 1:** Sample interview questions

**• Can you tell me about your experiences using RAI-based home care data?** Prompt: Do you feel confident in your ability to use these data sets? Why/why not?	
**• How is the home care data used for addressing safety issues?** Prompt: For individual clients? For your organization/region?	
**• How is the home care data used for implementing quality improvement initiatives within your organization?**	
**• Do you have any ideas on what materials should be available to home care managers and providers in using this data?**	

## Results

All of the participants we spoke with used some form of the interRAI assessment data for quality improvement in their organization. Participants discussed overseeing the implementation of RAI tools, providing structure and support for home care projects, understanding the data reports generated, and supervising quality improvement initiatives. Several participants provided powerful examples of how interRAI data have been used to guide patient safety and quality improvement initiatives; however, all participants recognized the challenges inherent in using these data.

### Utilizing interRAI data in home care

Participants explained how they presently use RAI data to guide and assess the performance of their home care organizations. In particular, the RAI data that are reported back to organizations give them a clear picture of how their organization is doing compared to others in their region:

*The information that we get from interRAI Canada is that they give us our [organization’s] data but they also compare it, other home care providers, generic of course, as a whole … And so it allows us to really look at, are we on par with other providers? And so that really helps us to identify as well the learning needs of our, of our assessors … You know are they on target, are we falling short, you know where can we improve sort of piece. So that’s been beneficial.* (Interview 2)

Other participants explained how RAI data helped them to see the “bigger picture” of their organization, as the provision of home care is rapidly changing and evolving. Having this big picture allows them to make crucial and timely business decisions:

*… there’s some really important business decisions that need to be made, and home care is something that is growing at a quicker rate funding-wise and the clients that we take care of than other areas within the healthcare system and understanding how our business is serving clients … . So there’s a whole bunch of different pieces around data that’s so important to us that really helps guide our zones and guide us provincially around where new money needs to be invested to continue to grow home care.* (Focus Group 2)

Finally, participants shared specific examples of how RAI data have helped them to identify, implement, and track several quality and safety improvement initiatives:

*We’re using the quality indicators that come out of the RAI-HC, running those on an annual basis to take a look at what are some of the quality issues that we could be tackling as a program. So using that kind of information, so for example, our pain management, having that clinical information and evidence to support that, we really could be doing better as a program in terms of pain management for our clients because that was something that was notable that came out of quality indicators. Or issues around incontinence, you know, various things like that, all prevention [We] just finished collaborating with CPSI on a virtual falls collaborative, and the RAI data was fundamental in that as well, it’s a key component of what we can monitor, prevalence of certain indicators in the program.* (Focus Group 1)

This participant went on to share another example:

*They give us extracts of the RAI assessment every quarter, the regions supply that to us and it’s de-identified. So once a quarter, we actually get our home care data that we can look at … One of the quality improvements is antipsychotic use without a diagnosis of psychosis. So now what we have done is we’re pulling that information of who is on an antipsychotic in home care. Then we can get home care looking at it and finding out, okay does this person really need, how did they get on meds and do they really need it*?

In spite of these strengths, however, the resounding theme in our interviews and focus groups was that most jurisdictions are experiencing limitations to their use of interRAI home care data.

### Challenging factors in the home care context

All participants discussed challenges that they face when utilizing interRAI data in the home care context, including: the general “newness” of these data in many jurisdictions; the sheer volume of data; limited capacity and resources to “make sense” of the data; and connectivity issues in rural areas. The overarching theme was that they simply were not, and did not know how to best interpret and utilize the data.

#### “Newness”

Participants noted that data use in home care is a newer concept, and one that is still evolving:

*Home care data is a newer thing. The home care environment is a more complex environment. There’s lots of provincial nuance from province to province … the environment and the models of care are continuing to evolve and change. So we don’t necessarily have what I would call a very stable service delivery and policy environment right now around … home care services in particular creates some challenges and a bit of a dynamic element to the data. What kind of data should we be collecting, the continuing of the updating of the RAI assessment tools and that. So, so there is still lots I think that fall in the place of that but we’re hoping over the next they say 5 to 10 years, we may have an improved and more efficient way of capturing some of the data off the front end.* (Focus Group 3)

#### Volume & capacity

With these new tools and datasets, participants are still figuring out how to utilize this information. One focus group participant noted, “*it’s a rich dataset and it can be a bit overwhelming.*” Home care providers are confronted with both issues of volume, and insufficient capacity to work with large datasets:

*I think you know, just the volume of data, especially on the service data, it’s just unbelievably massive, right? So were looking at, I think it’s about, I want to say a million records a week on the service side. So having the capacity to really look at that’s such a large dataset, is challenging.* (Focus Group 2)

Several participants noted that they have collected large amounts of data, but do not necessarily have the personnel or skills to use the data in a meaningful way:

*I think the other thing is you know we’ve amassed large databases, we have multiple different databases, we’ve got our clinical database, we have a lot of data at our fingertips and sometimes it is that overload with our clinicians, you know, we do still have a generation of, some generation of our workers and staff that don’t really see anything in numbers, and, I don’t want to say the touchy-feely type [laughs] but I’m a number person, I can see in the numbers quite a lot about a person, so it’s very hard to get some folks to see that as well.* (Focus Group 1)

*I think the core is the challenge for a lot of the providers is building the knowledge of what is available and then the capacity and the skills to use it, right? And to somehow embed it in quality improvement processes, etc. So it’s lots of tools but can also be a flood of data out there that, that doesn’t necessarily bring the value add.* (Focus Group 3)

#### Highly-trained personnel

The agencies that were able to use the data in ways that add value were the ones with highly trained personnel, individuals with PhD and or/extensive previous experience working with large clinical datasets, including interRAI data in other sectors. An example of a dedicated, highly-trained position is outlined below:

*So, one of the reasons that my position as a researcher was created here … in the home care program specifically was so that the program could mine that data more so than it had done previously. So as the data accumulated after bringing in the RAI-HC into the region, it became more and more clear to how important and useful that information was and that to really be able to leverage it for program planning and evaluation, policy development, decision-making, that they really needed someone who could work with the data and have that expertise that they didn’t currently have in the program. So that’s why my job was created, specifically because of my interRAI background.*

#### Agency size and location

The agencies most able to work with interRAI data were those with qualified, dedicated staff. Smaller agencies and those in rural areas had greater challenges working with these larger datasets:

*I also think that that bigger agencies have really fulsome quality departments and program evaluation and that kind of stuff. I don’t think that the smaller agencies or some of the smaller community support service agencies … I don’t think that they’ve got the kind, the quality staff or data collection … They report the number of clients served kind of thing.* (Interview 3)

Participants with clients in rural areas also experienced issues related to internet connectivity, which prevented their staff from completing interRAI assessments. Data were not being captured consistently:

*However, what is happening is, is because of spotty connectivity issues, our clinical staff are going in doing [RAI assessments] in hardcopy, returning to the office, then generating it, uh data entering it electronically … then returning back to the client’s home, to inform the care planning and at times are not returning back to the client home, just because of the, the increase to the work load … So that’s where we’re falling short.*

*… the connectivity challenges I know were, were definitely an issue across the board with all the providers that were involved in the collaborative and I think there was five other providers and they were all finding the same issue, so it’s not isolated to [our organization].* (Interview 2)

### Opportunities for education and capacity-building

Participants see the value and potential in the interRAI tools, but recognize that in most jurisdictions, in particular those without dedicated and highly-trained personnel, they are not able to optimally utilize the data. Participants are unfamiliar and insufficiently equipped to work with large data sets, feel overwhelmed by the volume of data, and do not have the right personnel to analyse the data in meaningful ways. Participants are using interRAI data, but feel that they are not using it to its fullest capacity. As one participant explained:

*I think we could do a much better job, absolutely. I do think what we’re doing is the bare minimum. I think that if there could be offered some regular lunch-and learns, or webinars, or conference calls, things of that nature.* (Interview 2)

When asked if she was confident in her ability to use the datasets, another participant explained:

*I would say we’re still in the learning process... but we’re starting to use the data. I would really like to see more push on, you know, now we’ve got the home care data, what do we do with it?*

In order to bolster their ability to utilize interRAI data, several participants noted that they are presently working with researchers at the Universities of Waterloo, Calgary and Alberta to examine particular outcome scales and other areas of interest. In addition to university partnerships, participants enthusiastically supported and appreciated the training modules provided by CIHI. For example, one participant commented:

*We have a good relationship with CIHI and so all of our case coordinators go through the CIHI education before, because that’s how they’re trained on doing the clinical assessments. We have our in-house specialists as well, but every new case coordinator does have to have the CIHI education and then that also imprints upon them the appropriate coding so that we have good quality data … . I really do believe CIHI has done an excellent job of doing those, providing those materials, having quick reference sheets about the outcome scores so that uh it is quick referral for them.* (Focus Group 1)

When probed, all participants requested additional data usage/health informatics training in online, webinar formats. One interviewee explained:

*Because home care is so spread out and, really, we hardly have enough time to breathe, I think webinars are the best … and webinars at times that are not work times. So doing it from noon to 1 doesn’t really help because, we don’t, nobody takes lunches.* (Interview 3)

## Discussion

It has been said that the provision of home care services in Canada is often based more on where a patient lives than what they actually need [13]; our findings indicate that the same can be said of home care data usage. The use of interRAI data appears to vary highly between provinces and jurisdictions, with some organizations utilizing the data better than others. In order to best serve the more than 2.2 million Canadians receiving home care services [14], the interRAI reporting system offers numerous opportunities to improve decision-making, patient safety and quality. Our participants shared several powerful examples of how these data have allowed them to appropriately intervene in the areas of medication/antipsychotics use, falls and pain management. This is consistent with research in LTC, which has demonstrated that interRAI data, when appropriately leveraged, can be used to maintain quality and inform policy decisions [15].

While participants recognized the power and potential of the interRAI data, they also emphasized a host of challenges that their home care organizations face if they are to use the data to its fullest potential. Most participants acknowledged that they are simply doing “*the bare minimum*”, largely because they are not equipped to interpret or utilize the data. InterRAI is relatively new to home care, while it is more established at other points of service delivery, such as LTC [16]. The overarching theme was “*we’ve got the home care data, what do we do with it?*” While some participants discussed specific barriers, such as connectivity issues in rural areas, the most common challenge was organizations feeling like they were insufficiently equipped to make sense of the data, to make it meaningful and useful for their organization. This is consistent with Prosperi and colleagues’ assertion that an abundance of data does not necessarily translate to the ability to use those data for targeted improvements in health services delivery [17]. The data must be matched with commensurate resources, training and personnel.

In Canada, we have seen that even medical students and physicians are not sufficiently trained in health informatics and how to appropriately assess and use large clinical datasets for care planning and quality improvement [18]. It is therefore not surprising that in the home care sector, in particular smaller agencies and frontline staff, personnel are also not receiving adequate education and support in the application of health informatics. Participants enthusiastically reported on valuable education that CIHI provides, but recognized that the training needs to go farther. Currently, this training is required for assessors who are using the tool at the frontline, but not for those who are using the datasets for quality improvement initiatives. At present, the organizations and jurisdictions that are more fully using the interRAI data are those with dedicated resources and highly-trained staff with either familiarity with the interRAI systems and/or previous exposure to large datasets. Our findings suggest that it is incumbent upon CIHI, university partners and the provinces that oversee the delivery of home care to further the educational opportunities for organizations that seek to use large datasets, such as interRAI, to guide decision-making and improve quality of care.

### Limitations

We recognize that not every province that collects and uses interRAI data for home care was represented in this sample; of the seven provinces in the CIHI database, we interviewed participants from five. Also, data were collected in 2016 and we acknowledge that advancements and training initiatives may have been made in the interim. Notwithstanding, our ongoing engagement with the home care sector in several provinces indicates that issues with data collection, interpretation and usage still present opportunities for improvement.

We note also that our focus in this paper is on data use by persons in management or data analysis positions; additional investigation is needed to explore use of interRAI data by home care clinicians.

## Conclusions

Fostering safety and quality improvements in home care is crucial, and poses unique challenges [19–21]. Mandated clinical datasets, such as the suite of interRAI tools, have the potential to improve quality and inform decision-making for the patient, and at a policy level (e.g., [15, 22, 23]). However, in order to utilize these data to their potential, home care agencies require commensurate education, personnel and supports. Our findings suggest that in the complex home environment, in particular in smaller and rural agencies, home care providers are ill-equipped and overwhelmed. These findings indicate a need for additional training and resources for these organizations, not only to support the agencies providing this vital service, but also to improve health outcomes for the millions of individuals in receipt of home care.

## Supplementary information

**Additional file 1.** Interview Guide.

## Data Availability

Data used in this analysis can be made available, upon reasonable request, by contacting the corresponding author, Dr. Paul Stolee (email: stolee@uwaterloo.ca).

## Abbreviations

CIHI: Canadian Institute for Health Information
CPSI: Canadian Patient Safety Institute
HC-QIs: Home care quality indicators
HCRS: Home Care Reporting System
interRAI-CHA©: interRAI Community Health Assessment
interRAI-HC©: interRAI Home Care Assessment
LTC: Long term care
