# Supplementary material for: “We’ve got the home care data, what do we do with it?”: understanding data use in decision making and quality improvement
Source: BMC Health Serv Res. 2020 Mar 25;20:251. doi: 10.1186/s12913-020-5018-9 (PMC7093944; doi:10.1186/s12913-020-5018-9)
Supplement: Supplementary file 1 — Additional file 1. Interview Guide. [file 12913_2020_5018_MOESM1_ESM.docx]

**Interview Guide**

*Introduction to purpose of the project, review Letter of Information and obtain consent (verbal and written).*

**Section 1: CONTEXTUAL INFORMATION**

1. Can you please describe your role to me?

**Section 2: OPEN-ENDED, EXPERIENCES USING DATA SETS**

1. Can you tell me about your experiences using home care data (both RAI-based data and other locally collected data)?
   1. How confident do you feel in your ability to use these data sets?
   2. Are there reasons that you feel confident (or not confident) utilizing this information?
   3. Are you able to easily access the home care data? Why or why not?
2. How is the home care data used for **addressing safety issues**?
   1. For individual clients?
   2. For your organization/region?
3. How is the home care data used for **implementing quality improvement initiatives** within your organization?
4. Do you think that there is anything that can be done to increase your confidence and use of these data sets?
   - IF YES: What would be beneficial for your use?

**Section 3: OPEN-ENDED, EDUCATION IDEAS**

1. Can you please tell me about the resources, materials, or education that you have received for using home care data collected through the RAI or other locally collected data?
2. Are you aware of any educational materials or resources that are available for using home care data?
   1. If yes, can you tell me how you found this information?
   2. Do you feel this information helped you in using home care data for QI purposes?
3. Do you have any ideas or suggestions for what materials should be available to home care managers and providers to assist in using this data for QI purposes?
   1. How do you believe this information would aid in addressing safety issues or implementing quality improvement initiatives?
   2. How would you disseminate this information so that all providers using the data have access to these resources?

**Section 4: ENDING QUESTIONS**

1. Is there anything else you think I should know?
